# Genomic and Transcriptomic Associations Identify a New Insecticide Resistance Phenotype for the Selective Sweep at the *Cyp6g1* Locus of *Drosophila melanogaster*

**DOI:** 10.1534/g3.116.031054

**Published:** 2016-06-15

**Authors:** Paul Battlay, Joshua M. Schmidt, Alexandre Fournier-Level, Charles Robin

**Affiliations:** The Bio21 Institute and the School of Biosciences, The University of Melbourne, Parkville, Victoria 3010, Australia

**Keywords:** azinphos-methyl, *D. melanogaster*, *Drosophila* Genetic Reference Panel, systems genetics, *Cyp6g1*

## Abstract

Scans of the *Drosophila melanogaster* genome have identified organophosphate resistance loci among those with the most pronounced signature of positive selection. In this study, the molecular basis of resistance to the organophosphate insecticide azinphos-methyl was investigated using the *Drosophila* Genetic Reference Panel, and genome-wide association. Recently released full transcriptome data were used to extend the utility of the *Drosophila* Genetic Reference Panel resource beyond traditional genome-wide association studies to allow systems genetics analyses of phenotypes. We found that both genomic and transcriptomic associations independently identified *Cyp6g1*, a gene involved in resistance to DDT and neonicotinoid insecticides, as the top candidate for azinphos-methyl resistance. This was verified by transgenically overexpressing *Cyp6g1* using natural regulatory elements from a resistant allele, resulting in a 6.5-fold increase in resistance. We also identified four novel candidate genes associated with azinphos-methyl resistance, all of which are involved in either regulation of fat storage, or nervous system development. In *Cyp6g1*, we find a demonstrable resistance locus, a verification that transcriptome data can be used to identify variants associated with insecticide resistance, and an overlap between peaks of a genome-wide association study, and a genome-wide selective sweep analysis.

Genome-wide scans for positive selection have become possible over recent years, and reveal fascinating insights into recent evolution, with a global perspective afforded by whole genome analyses. These scans are becoming increasingly sophisticated as methods advance from a focus on hard sweeps to partial sweeps and soft sweeps. Whereas a locus with a hard sweep has a single haplotype surrounding a single adaptive variant, a locus with a soft sweep has multiple haplotypes containing one or more selected variants. Partial sweeps occur when adaptive variants have not reached fixation. Studies such as these lead to candidates of selection in a completely unbiased way; however, it is not always easy to deduce what selective force is driving the selection on identified genes, and the lack of phenotypic validation of candidates has been a major criticism of these approaches (Jensen *et al.* 2016). Resistance to insecticides is a compelling evolutionary model, due to the relatively recent introduction of these toxins, and the specific selective pressures they are capable of imparting. This model has, however, tended to focus on genes of major effect. The *Drosophila* Genetic Reference Panel (DGRP), a set of inbred *Drosophila melanogaster* lines with sequenced genomes and transcriptomes (Mackay *et al.* 2012; Huang *et al.* 2015), allows for the identification of both major and minor effect alleles contributing to resistance phenotypes, in the context of recent selection.

In 2015, Garud *et al.* identified regions of the *D. melanogaster* genome under strong, recent selection by interrogating the sequences of DGRP lines for signatures of selective sweeps (Garud *et al.* 2015). The top three regions identified in this screen, *Cyp6g1*, *Ace*, and *CHKov1* had all been previously associated with resistance to insecticides (Daborn *et al.* 2001; Pralavorio and Fournier 1992; Aminetzach *et al.* 2005), and two of them to a particular insecticide: the organophosphate (OP) azinphos-methyl.

Resistance to OPs is arguably the best understood of any resistance to an insecticide class. Widespread use of OPs for more than half a century on a range of pests has resulted in many well-studied cases of resistance to members of this class of toxin (Siegfried and Scharf 2001). Acetylcholinesterase (Ace) is the molecular target of OPs. Bound in the postsynaptic membrane, it hydrolyses the ester bond in acetylcholine following neurotransmission, ending the signal. OPs bind irreversibly to Ace, causing a build-up of acetylcholine in the synapse, and continuous stimulation of the postsynaptic neuron. This results in paralyzing seizures, and the eventual death of the insect. Four substitutions in *D. melanogaster* Ace cause insensitivity of the enzyme to OPs. These mutations occur together in some alleles, in many cases acting cooperatively to increase resistance, with differing combinations maximizing resistance to different insecticides by either restricting access or affecting the position of key catalytic residues (Mutero *et al.* 1994; Menozzi *et al.* 2004). Additionally, duplications of *Ace* exhibit extreme population differentiation (Kolaczkowski *et al.* 2011), providing further evidence that selection is acting at this locus in *D. melanogaster*.

Another of Garud *et al.*’s top three candidate genes is *CHKov1*, originally identified in a screen of *D. melanogaster* transposable element polymorphisms under recent, positive selection (Aminetzach *et al.* 2005). The same study then linked the *CHKov1-DOC* allele (containing the insertion of *doc1420* into the coding region of this uncharacterized gene) with resistance to the OP azinphos-methyl by comparing two strains differing by a single introgressed region. In 2011, Magwire *et al.* reported that resistance to the sigma virus Rhabdoviridae mapped to a region containing *CHKov1*, a result that was supported using a genome-wide association study (GWAS) in the DGRP population (Magwire *et al.* 2011).

A cytochrome P450 gene, *Cyp6g1*, is also one of Garud *et al.*’s top three candidates for positive selection (Garud *et al.* 2015). Naturally occurring alleles causing the overexpression of *Cyp6g1* result in resistance to DDT and neonicotinoids (Daborn *et al.* 2001), which is attributable to *Cyp6g1*-limited metabolism of these toxins (Joussen *et al.* 2008; Hoi *et al.* 2014). Resistance to the OP diazinon in Australian populations was mapped to a region containing *Cyp6g1* (Pyke *et al.* 2004). Daborn *et al.* (2007) subsequently reported, however, that transgenic *Cyp6g1* overexpression was incapable of conferring resistance to diazinon.

Here, we describe a systems genetics approach (Ayroles *et al.* 2009) that incorporates into a single model associations of phenotypic, genomic, and transcriptomic variation to investigate resistance to azinphos-methyl using the DGRP population. This study aimed to characterize resistance to this insecticide from a polygenic framework, with the added advantage of being able to assess the involvement of the peaks identified by selective sweep analysis in azinphos-methyl resistance, using the DGRP population in which they were detected.

## Materials and Methods

### Fly lines

The DGRP lines examined in this study were generated by Mackay *et al.* (2012), and were obtained from the Bloomington Drosophila stock center in Indiana. *6g1*HR-GAL4, UAS-*Cyp6g1* and Phi86 lines were generated by Chung *et al.* (2007). All fly stocks were maintained at 25° on rich medium containing, maltose (46 g/L), dextrose (75 g/L), yeast (35 g/L), soy flour (20 g/L), maize meal (73 g/L), agar (6 g/L), acid mix (14 ml/L), and tegosept (16 ml/L). The acid mix solution was made up of orthophosphoric acid (42 ml/L), and propionic acid (412 ml/L), while the tegosept solution was 50 g tegosept dissolved in 950 ml of 95% EtOH. Applicable quantities of azinphos-methyl were mixed into rich medium once it had cooled below 60°, to produce insecticide media.

### Insect bioassays

First-instar larvae (< 24 hr old) were collected from laying plates and transferred onto insecticide media at a density of 20 larvae per vial. Controls were performed using media containing no insecticide. The number of fully formed pupae were scored after 7 d. Three biological replicates were performed for each dose.

### Calculation of LD_50_

For each DGRP line, dose data were corrected for control mortality using Abbott’s correction, and linear models were fitted to dose-mortality data on a log-probit scale using ‘glm’ in the R statistical package (R Core Team 2015) and scripts from (Johnson *et al.* 2013). 50% lethal dose (LD_50_) values and 95% confidence intervals were calculated using Fieller’s method from fitted linear models (Finney 1971).

### Genome-wide association studies

**Figure 1:**
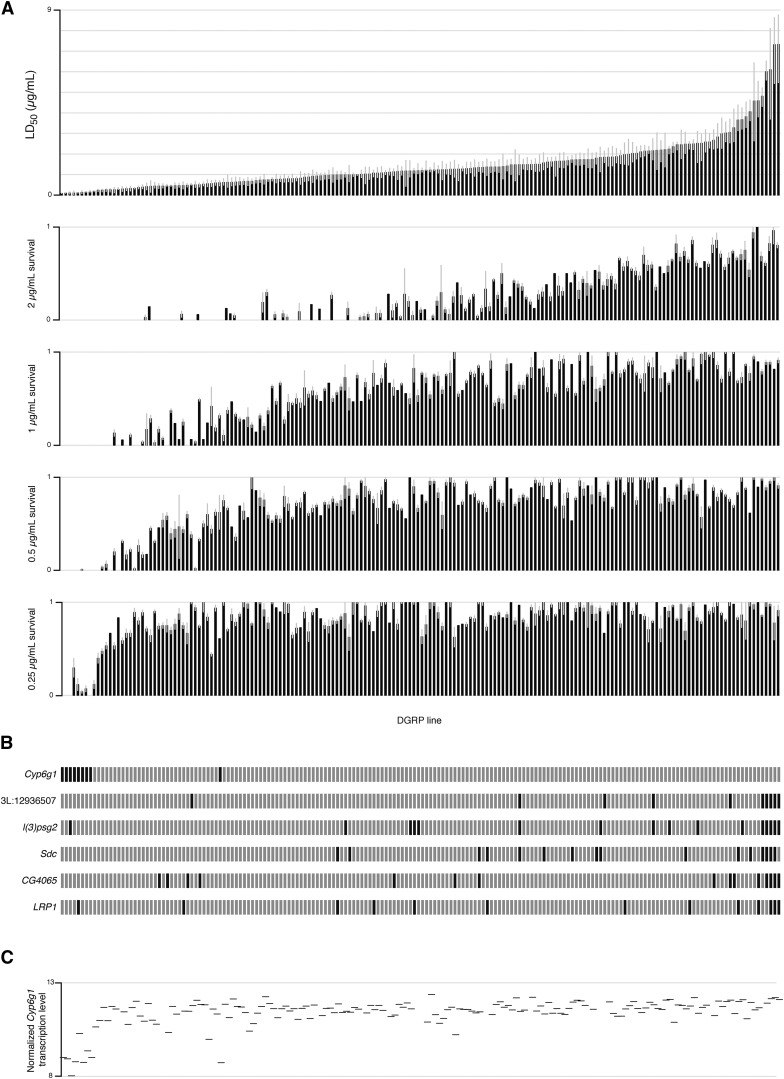
(A) Azinphos-methyl LD_50_ phenotype (error bars represent 95% C.I.) and four mean azinphos-methyl survival phenotypes at single doses (error bars represent SEM) for 178 DGRP lines, ordered by LD_50_ phenotype. (B) Lines carrying minor allele (black) of GWAS candidates. (C) Mean of male and female normalized *Cyp6g1* transcription level as measured by Huang *et al.* (2015), data missing for some lines.

Phenotypes for 178 lines at each of the four common doses, and the LD_50_, were submitted to the Mackay Lab DGRP2 pipeline as five separate GWAS (Figure 1A; http://dgrp.gnets.ncsu.edu/; Huang *et al.* 2014).

### In silico genotyping

*y*; *cn bw sp*; assembled reference genome sequence version 5.33 was recovered from FlyBase (Millburn *et al.* 2016). DGRP line sequences from Illumina platforms were obtained from the Baylor College of Medicine website (https://www.hgsc.bcm.edu/content/dgrp-lines; Mackay *et al.* 2012). Reads were aligned to the *y*; *cn bw sp*; reference genome using Burrows-Wheeler Aligner (Li and Durbin 2009). Alignments of Illumina paired end reads to the *y*; *cn bw sp*; genome in regions containing *CHKov1* and *Cyp6g1* loci were analyzed with IGV 2.0 software (Robinson *et al.* 2011) to score structural variation and transposable element presence in each line. Alignments were used to identify and plot DGRP variation at each base in exons III and IV of *Ace*.

### Preparation of transcriptome data

Transcriptome data for 1- to 3 d old adult flies from 185 DGRP lines were recovered from the DGRP website (http://dgrp.gnets.ncsu.edu/data.html; Huang *et al.* 2015). Mean transcription level was calculated for each gene from two biological replicates, for each of the 18,140 transcripts measured by Huang *et al.* (2015) for each sex and in each DGRP line.

### Structural equation modeling

The ‘sem’ package (Fox *et al.* 2014) in R (R Core Team 2015) was used to generate a structural equation model incorporating factors associated below Bonferroni significance with azinphos-methyl resistance:

The six significantly associated single nucleotide polymorphisms (SNPs) from the LD_50_ GWAS as fixed variables.
The *Cyp6g1-M* allele identified by significantly associated SNPs from the 0.25 and 0.5 µg/ml survival phenotype GWAS as a fixed variable.
Expression of *Cyp6g1* and *Cyp6g2* (mean of male and female values) as random variables.
The azinphos-methyl LD_50_ phenotype as a random variable.

### Cyp6g1 overexpression

*Cyp6g1* overexpression using the GAL4/UAS system (Brand and Perrimon 1993) was originally described by Chung *et al.* (2007). *6g1*HR-GAL4 females, in which GAL4 is regulated by *Cyp6g1* upstream sequence originating from Hikone-R line flies, were crossed to UAS-*Cyp6g1* males, which carry an additional copy of *Cyp6g1* coding region under control of a UAS promoter. In the control cross Phi86 line males were used, which contain the UAS promoter but lack the additional *Cyp6g1* coding region downstream.

### Data availability

Strains are available upon request. Supplemental Material, File S1 contains detailed descriptions of all supplemental files. File S2 contains phenotypes for all five GWAS. Figure S1 contains plots of *Cyp6g1* transcription level against LD_50_ phenotype. Figure S2 contains details of DGRP *Ace* variation in exons III and IV.

## Results

### GWAS of resistance to azinphos-methyl

A total of 178 DGRP lines was assayed for survival to pupation on rich medium containing azinphos-methyl at 0, 0.25, 0.5, 1, and 2 µg/ml, with additional doses (ranging from 0.0625 to 8 µg/ml) used to quantify the LD_50_ of lines with extreme phenotypes. LD_50_ values were calculated from probit models fit to survival data (corrected for control mortality using Abbott’s correction) from each line at each dose, and ranged from 0.083 µg/ml to 7.33 µg/ml. Phenotypes for 178 lines at each of the four common doses, and the LD_50_, were submitted to the Mackay Lab DGRP2 pipeline as five separate GWAS (Figure 1A; http://dgrp.gnets.ncsu.edu/; Huang *et al.* 2014).

**Figure 2:**
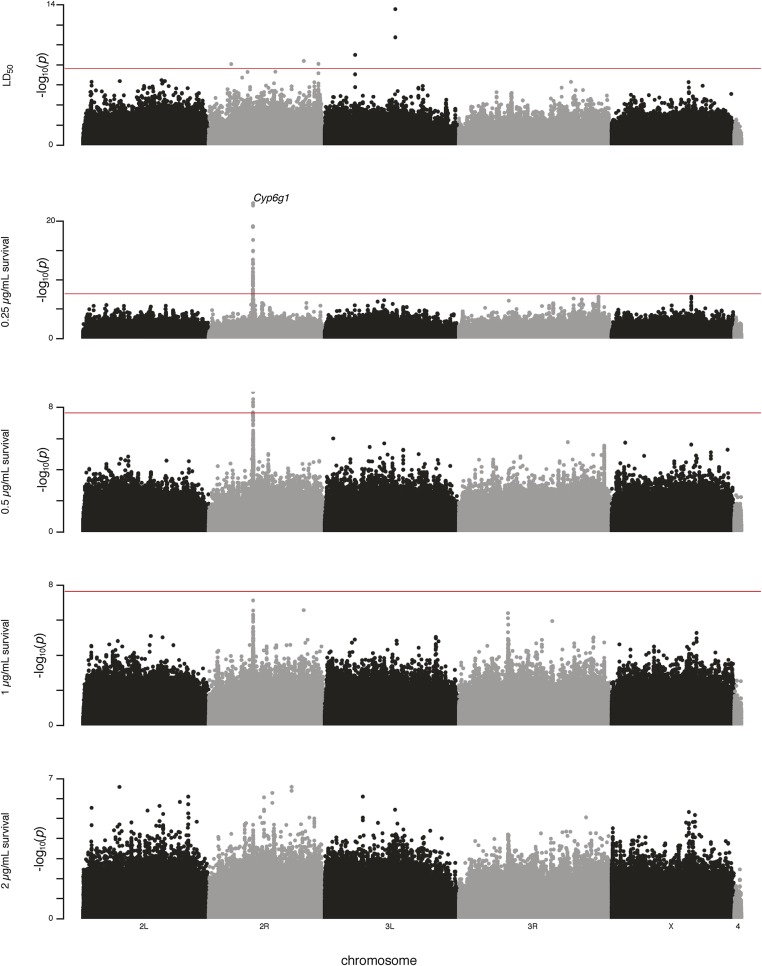
Manhattan plots for GWAS of LD_50_, 0.25 µg/ml survival, 0.5 µg/ml survival, 1 µg/ml survival, and 2 µg/ml survival azinphos-methyl phenotypes. The *x*-axis shows genomic location of variant, the *y*-axis shows –log_10_(*P*-value of association with phenotype). Bonferroni threshold for genome-wide significance (2.28 × 10^−8^) is shown.

Three of the five GWAS were able to identify phenotype-associated SNPs with *P*-values below the Bonferroni threshold for genome-wide significance (2.28 × 10^−8^; Figure 2). Considering results from all five GWAS, the strongest association (*P* = 6.6 × 10–24) is from the 0.25 µg/ml survival phenotype, and is located in an intron of *Cyp6g1*. All significant SNPs (below the Bonferroni threshold) in GWAS for both 0.25 µg/ml survival and 0.5 µg/ml survival phenotypes are in this same ∼70 kb region centered around *Cyp6g1* (Table 1 and Figure 2). The three most significant *Cyp6g1* SNPs are present in nine DGRP lines, eight of which are extremely susceptible to azinphos-methyl (Figure 1B). *In silico* genotyping methods reveal these nine lines to be the only DGRP lines that are homozygous for *Cyp6g1-M*—the ancestral allele of *Cyp6g1*—and the most susceptible to DDT (Schmidt *et al.* 2010).

**Table 1:** Variants with *P*-values below the Bonferroni threshold for genome-wide significance (2.28 × 10^−8^) from GWAS of five azinphos-methyl phenotypes

Phenotype	Candidate	Site Class	No. Variants	Location	Minimum *P*-Value
0.25 µg/ml survival	*Cyp6g1*	Various	45	2R:12131954-2R:12202171	6.579 × 10^−24^
0.5 µg/ml survival	*Cyp6g1*	Various	8	2R:12185332-2R:12202171	1.02 × 10^−9^
LD_50_	Unannotated	Intergenic	2	3L:12936507-3L:12936514	2.62 × 10^−14^
LD_50_	*l(3)psg2*	Nonsynonymous	1	3L:5586237	9.93 × 10^−10^
LD_50_	*Sdc*	Intronic	1	2R:21457715	3.98 × 10^−9^
LD_50_	*CG4065*	Synonymous	1	2R:24135649	7.80 × 10^−9^
LD_50_	*LRP1*	Intronic	1	2R:8191283	8.15 × 10^−9^

Multiple variants indicating a single region are grouped together.

SNPs in and around *Cyp6g1* were not detected by the LD_50_ GWAS, which identified instead six other Bonferroni-significant SNPs (Table 1).

### Phenotype to transcriptome associations

A linear model was fit between azinphos-methyl LD_50_ values from 159 DGRP lines, and mean transcription level of each gene measured by Huang *et al.* (2015). Of the 18,140 transcripts in this dataset, a single transcript for each sex was found to be associated with the phenotype with a *P*-value below the Bonferroni threshold for transcriptome-wide significance (2.76 × 10^−6^). In the case of both male and female associations, this transcript mapped to *Cyp6g1* (*P* = 1.93 × 10^−6^, *P* = 2.75 × 10^−7^ respectively; Figure 1C and Figure S1). Transcriptome associations with the four single-dose phenotypes yielded similar results (data not shown). This supports the finding from our GWAS that alleles of *Cyp6g1*, which have been demonstrated to increase transcription level and hence resistance to DDT, imidacloprid and nitenpyram (Daborn *et al.* 2001, 2002, 2007; Schmidt *et al.* 2010), are associated with resistance to azinphos-methyl in DGRP lines.

### Structural equation model

**Figure 3:**
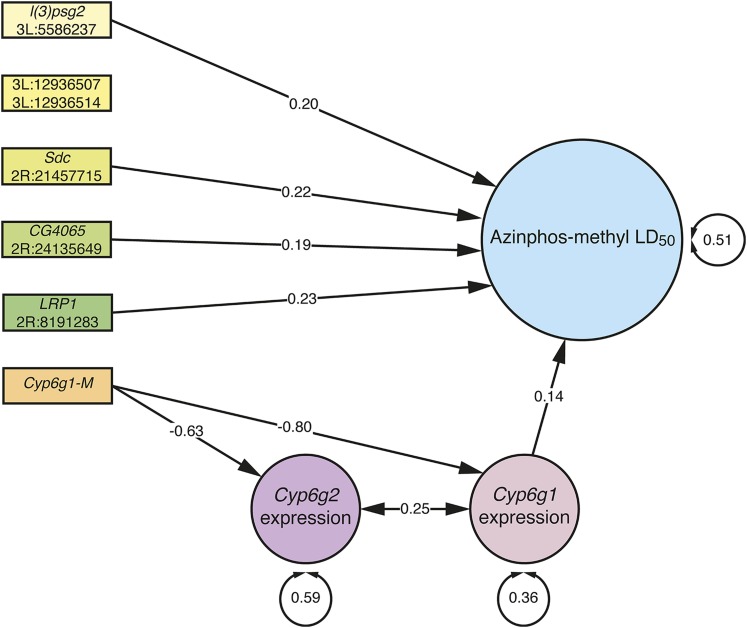
Structural equation model showing the influence of Bonferroni-significant factors from GWAS, and transcriptome-phenotype associations in the azinphos-methyl LD_50_ phenotype. Standardized coefficients are shown on paths; only statistically significant (*P* < 0.05) paths are shown. Standardized coefficients account for substitution of homozygous major allele by homozygous minor allele. The involvement of 3L:12936507 and 3L:12936514 SNPs was rejected by the model.

Structural equation modeling was used to test the involvement of Bonferroni-significant factors from GWAS and transcriptome-phenotype associations in the azinphos-methyl LD_50_ phenotype (*Cyp6g2* expression level was included due to its correlation with *Cyp6g1* expression), and the model explained the data significantly well (χ^2^ = 6.83, d.f. = 10, *P* = 0.74; Figure 3). The model did not show a significant influence by two SNPs (3L:12936507 and 3L:12936514), but supported the influence of the other four Bonferroni-significant SNPs on the phenotype, and showed their involvement was independent of *Cyp6g1*, as no systematically significant path was found connecting these SNPs to the phenotype indirectly, through *Cyp6g1* expression. Systematically significant paths were found connecting the *Cyp6g1-M* allele to expression of both *Cyp6g1* and *Cyp6g2*, but only *Cyp6g1* expression was found to have a significant influence on the phenotype.

### Verification of Cyp6g1 as an azinphos-methyl resistance mechanism

**Figure 4:**
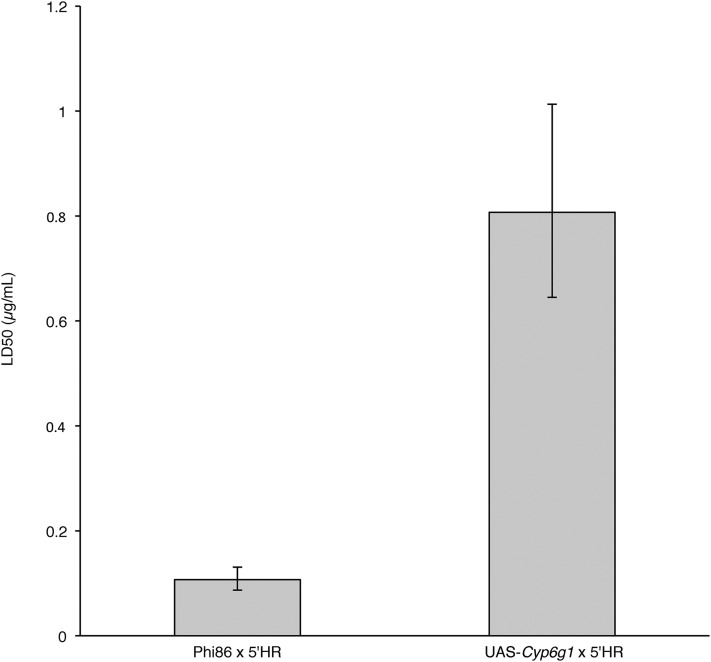
Azinphos-methyl LD_50_ of *Cyp6g1*-overexpression flies (UAS-*Cyp6g1* × 5′HR) compared with the relevant control (Phi86 × 5′HR). Error bars represent 95% C.I.

Flies transgenically overexpressing *Cyp6g1* using the GAL4-UAS system, driven by upstream elements from a DDT-resistant *Cyp6g1* allele (Chung *et al.* 2007), were phenotyped on azinphos-methyl laced media. The LD_50_ of these flies was significantly higher, and 6.5-fold greater, than controls that did not overexpress the enzyme (Figure 4).

### Cyp6g1-AA and Cyp6g1-BA alleles

**Figure 5:**
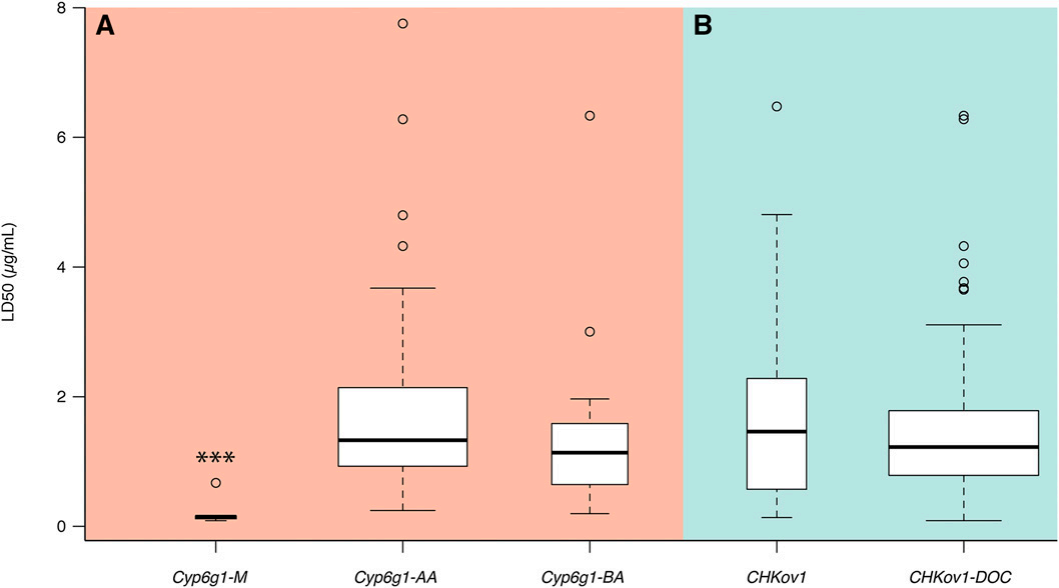
Mean azinphos-methyl LD_50_ phenotypes for structural variants at (A) *Cyp6g1*, and (B) *CHKov1*. Note there is significant difference in mean LD_50_ between *Cyp6g1-AA* and *Cyp6g1-BA* alleles, or between *CHKov1* and *CHKov1-DOC* alleles (Student’s *t*-test; *P* > 0.05).

DDT-resistant *Cyp6g1-AA* and *Cyp6g1-BA* alleles are both present in the DGRP. *Cyp6g1-BA* has been shown to confer tissue-specific expression differences, and a slight increase in male DDT resistance, over *Cyp6g1-AA* (Schmidt *et al.* 2010). We find no significant difference between the mean azinphos-methyl LD_50_ values for each of these alleles (Figure 5A).

### CHKov1 alleles

It was previously reported that insertion of the *doc1420* transposable element into the coding region of *CHKov1* increases resistance to azinphos-methyl (Aminetzach *et al.* 2005). DGRP lines were genotyped for this structural variation, and the mean azinphos-methyl LD_50_ for each class was compared. There was no significant difference identified between the groups (Figure 5B).

### Ace resistance substitutions in the DGRP

Menozzi *et al.* (2004) identify four common substitutions near the active groove of Ace that reduce sensitivity to various organophosphate and carbamate insecticides. Analysis of DGRP sequence data reveals that three of these four substitutions (I161V, G265A and F330Y) are polymorphic in the DGRP at moderate frequencies, while one (G368A) is entirely absent (Figure S2).

## Discussion

### Cyp6g1

Here we have shown that the strongest genome-wide association detected out of five azinphos-methyl resistance phenotypes (four single doses and the LD_50_) identifies *Cyp6g1*—a gene previously associated with resistance to insecticides. *Cyp6g1* was first described as a DDT resistance gene by Daborn *et al.* (2001), who found that DDT-resistant lines of *D. melanogaster* contain an *Accord* transposable element insertion upstream of the gene (Daborn *et al.* 2002), which correlates with increased *Cyp6g1* expression. Chung *et al.* (2007) showed this increased expression to be in specific tissues, important for insecticide detoxification. *Cyp6g1* cross-resistance was additionally described to the neonicotinoids imidacloprid (Daborn *et al.* 2001) and nitenpyram (Daborn *et al.* 2007), and, in 2008, the capacity of the enzyme to metabolize both DDT and imidacloprid was demonstrated in cell culture by Joussen *et al.* (2008).

Four alleles of *Cyp6g1* were described by Schmidt *et al.* (2010); the previously identified *Cyp6g1-Accord* allele was found to also involve a tandem duplication of the gene (*Cyp6g1-AA*), and two additional resistant alleles were described, characterized by two successive transposable element insertion events (*Cyp6g1-BA* and *Cyp6g1-BP*). The most derived of these, *Cyp6g1-BP*, is also the most DDT-resistant; however, it is absent from the DGRP. Both *Cyp6g1-AA* and *Cyp6g1-BA* confer resistance to DDT relative to the ancestral *Cyp6g1-M* allele, but the work of Schmidt *et al.* (2010) suggests this to be the smallest step, phenotypically, of the allelic series. Significant differences between *Cyp6g1-AA* and *Cyp6g1-BA* alleles were shown in DDT LD_50_ for males but not females, and in expression in the midgut but not the fat body. We found no difference between the mean azinphos-methyl LD_50_ values of *Cyp6g1-AA* and *Cyp6g1-BA* alleles in the DGRP (Figure 5A), which, given the subtleties in the phenotypes identified by Schmidt *et al.* (2010), is not surprising.

*Cyp6g1* was also associated with resistance to azinphos-methyl by comparing the LD_50_ phenotype to transcriptome data from 185 DGRP lines gathered by Huang *et al.* (2015). While this is consistent with our findings that alleles increasing *Cyp6g1* expression are associated with resistance, it also provides further evidence that candidate genes may be identified by associations between phenotype and transcriptome. This supports the work of Ayroles *et al.* (2009), who found, using the original 40 DGRP transcriptomes, that verifiable associations can be detected between phenotype and transcription level. This additional dimension to the analysis of the molecular basis of phenotypic variation in the DGRP should prove more powerful when the phenotype used matches the transcriptome data of Huang *et al.* (2015), specifically by sex and lifestage.

Comparing transcription level directly with a phenotype is powerful, as it relies on the measurement of a functionally relevant attribute. Thus, evolutionary unrelated variants can be pooled together based on transcription level, thereby alleviating the issue of allelic heterogeneity that can confound GWAS. This may be especially significant when the variants that are pooled are too rare to be picked up by GWAS.

Validation that increased *Cyp6g1* expression confers increases in azinphos-methyl resistance comes from our finding that transgenic overexpression of *Cyp6g1*, using the GAL4-UAS system and regulatory elements from the *Cyp6g1-AA* allele, is sufficient to confer a 6.5-fold increase in LD_50_. While we may speculate that this is due to improved metabolism of the insecticide by increased Cyp6g1 enzyme concentration in metabolic tissues, the ability of Cyp6g1 to metabolize azinphos-methyl remains to be demonstrated, as in the case of DDT and imidacloprid (Joussen *et al.* 2008; Hoi *et al.* 2014).

OP resistance has previously been linked to the chromosomal region containing *Cyp6g1*. Ogita (1958) described dominant cross-resistance between DDT and parathion. Kikkawa (1961) then mapped parathion resistance in the Hikone-R strain to a region on chromosome 2 also associated with DDT resistance, and also described cross-resistance to malathion. Pyke *et al.* (2004) mapped diazinon resistance to this same region, and found evidence of what Schmidt *et al.* (2010) would later describe as *Cyp6g1-AA* and *Cyp6g1-BP* alleles among resistant individuals. The findings of Pyke *et al.* (2004) were seemingly contradicted, however, by Daborn *et al.* (2007), who found transgenic overexpression of *Cyp6g2*, but not *Cyp6g1*, sufficient to confer diazinon resistance. The DGRP transcriptome data (Huang *et al.* 2015) demonstrates that expression of *Cyp6g1* is correlated with that of its tandem paralog *Cyp6g2* (*R*^2^ = 0.52 and 0.44 for male and female adults, respectively). So one tentative hypothesis is that diazinon resistance was mapped to *Cyp6g1* in a natural population due to the collateral upregulation of *Cyp6g2* in natural resistance alleles, which explains why transgenic overexpression of *Cyp6g1* alone failed to confer resistance. Our findings of azinphos-methyl resistance in this study differ from those with diazinon, as we were able to verify that *Cyp6g1* alone is capable of conferring high levels of resistance when transgenically overexpressed. While we do not know the capacity of overexpressed *Cyp6g2* to confer resistance to azinphos-methyl, structural equation model analysis suggests that *Cyp6g2* expression does not independently influence LD_50_ in DGRP lines (Figure 3).

### LD_50_ GWAS candidates

Although a verifiable azinphos-methyl resistance mechanism, *Cyp6g1* was identified by only two of the four single-dose GWAS, and not the LD_50_ GWAS. This demonstrates that the genetic architecture of related phenotypes, like a range of doses of the same insecticide, may vary significantly. In contrast to *Cyp6g1*, the six SNPs identified by the LD_50_ GWAS with *P*-values below the Bonferroni threshold (Table 1) are all low frequency variants enriched among resistant individuals (Figure 1B). Although integrated haplotype scores give no indication that these variants are under recent selection (data not shown), their identification may be informative of the biology of azinphos-methyl toxicity. Structural equation modeling supports the influence of four of these six SNPs on the LD_50_ phenotype, as factors independent of *Cyp6g1* expression (Figure 3).

A nonsynonymous SNP in the second exon of *lethal (3) persistant in salivary gland 2* (*l(3)psg2*) is predicted to cause a serine to threonine substitution at amino acid 726 of the protein. *l(3)psg2* is expressed in response to ecdysone, and involved in regulation of programmed cell death in the salivary glands during metamorphosis (Wang *et al.* 2008; Ihry and Bashirullah 2014). Although its role in the salivary gland has been specifically studied, *l(3)psg2* is expressed in a range of tissues, most highly, in larvae, in the central nervous system (Celniker *et al.* 2009; Chintapalli *et al.* 2007).

Syndecan (Sdc) is a heparin sulfate proteoglycan that is involved in axon guidance in central nervous system development by facilitating Slit-Robo binding (Chanana *et al.* 2009), and also in neuromuscular junction morphogenesis (Johnson *et al.* 2006). These functions of Sdc are reflected in its high expression in the larval central nervous system (Celniker *et al.* 2009; Chintapalli *et al.* 2007). However, Sdc is expressed in larvae at higher levels in the fat body (Chintapalli *et al.* 2007), where natural *Sdc* alleles have been found to affect variation in fat storage (De Luca *et al.* 2010). Given that azinphos-methyl binds its target in the neuromuscular junction, and exerts its effect through the nervous system, the development of these systems could certainly be involved in differences in sensitivity to the insecticide. Also intriguing in relation to insecticide resistance is Sdc’s involvement in fat storage, as the fat body is a key metabolic tissue, and, in fact, one of the tissues in which *Cyp6g1* is upregulated in resistant alleles (Chung *et al.* 2007). Fat storage is also relevant given the ultimate cause of death due to azinphos-methyl toxicity is likely to be a depletion of energy supplies.

Little is known about the function of CG4065 in *D. melanogaster*. It contains a region homologous with the Mak10 subunit of the NatC complex, shown in Zebrafish to be developmentally controlled, and required for cell proliferation and vessel formation in early development (Wenzlau *et al.* 2006). It is expressed in a range of larval tissues, but most highly in the central nervous system (Chintapalli *et al.* 2007).

*LDL receptor protein 1* (*LRP1*) is expressed in most cell types, but is highest in hepatocyte-like cells and neurons (Herz and Bock 2002). Its role in hepatocytes has been characterized in its mouse homolog, where it functions as a receptor for lipoproteins that carry lipids from the gut to the liver (Rohlmann *et al.* 1998). In the *D. melanogaster* brain, it has been demonstrated to facilitate transport across the blood–brain barrier of lipoprotein LTP, in order to regulate insulin-like peptide production in response to dietary lipid intake (Brankatschk *et al.* 2014). The role of LRP1 as a blood–brain barrier transporter is of particular interest in reference to azinphos-methyl, given the insecticide must enter the central nervous system to exert its effect. LRP1 was also identified in a previous DGRP GWAS of a food intake phenotype (Garlapow *et al.* 2015), with RNAi verification demonstrating *LRP1* knockdown significantly increases food uptake in males.

### CHKov1

Insertion of a *doc1420* transposable element into the coding region of *CHKov1* has previously been associated with resistance to azinphos-methyl in a single, introgressed *D. melanogaster* line (Aminetzach *et al.* 2005). More recently, Magwire *et al.* (2011) found, through linkage mapping and a subsequent DGRP GWAS, that the *CHKov1-DOC* allele was associated with resistance to the Sigma virus. Given that Magwire *et al.* (2011) were able to detect *CHKov1-DOC* in their GWAS from a haplotype of SNPs in linkage disequilibrium with the insertion, we may have expected to find the same haplotype significantly associated in any of our azinphos-methyl GWAS. To verify that *CHKov1-DOC* is not associated with this phenotype, we genotyped DGRP lines for the insertion and found no significant difference between LD_50_ values of lines carrying ancestral or *CHKov1-DOC* alleles (Figure 5B). In this study we found no evidence to support the involvement of *CHKov1* in resistance to azinphos-methyl, although we cannot rule out its effect on resistance in the adult life stage—the stage at which Aminetzach *et al.* (2005) performed toxicology studies.

### Ace

Another expected resistance mechanism, absent from our GWAS results, is variation in the target site of OP insecticides, Ace. Four substitutions in Ace have been demonstrated, *in vitro*, to affect binding of azinphos-methyl and other insecticides to the enzyme (Menozzi *et al.* 2004). In their genotyping of the four insensitivity substitutions in Ace alleles worldwide, Karasov *et al.* (2010) identified three substitutions at moderate frequencies, but found the fourth, G368A, absent. We found a similar pattern in DGRP genotypes, with G368A likewise absent (Figure S2). According to the binding kinetics analysis of Menozzi *et al.* (2004), G368A is required for high levels of Ace insensitivity to azinphos-methyl, and, although combinations of substitutions present in the DGRP are capable of reducing Ace sensitivity by as much as 4.3-fold, we do not see significant differences in mean LD_50_ values of lines grouped by substitution haplotype (data not shown). The insensitivity to azinphos-methyl by Ace in the DGRP is relatively small, given the spectrum of insensitivities achieved by ‘resistant’ Ace substitution haplotypes containing G368A, which are as high as 77-fold for azinphos-methyl (Menozzi *et al.* 2004).

### Conclusions

In this study, we utilized a systems genetics approach to uncover the molecular basis of resistance to azinphos-methyl—a strong candidate for a selective agent in the DGRP population according to a recent selective sweep scan. We find no evidence to support the involvement of *CHKov1-DOC* in resistance to azinphos-methyl, and we find that, although insecticide-resistant *Ace* alleles are present in the DGRP, alleles conferring high levels of insensitivity to azinphos-methyl are absent. However, we detect strong associations between our azinphos-methyl phenotype and both genomic and transcriptomic DGRP data, indicating that alleles of *Cyp6g1*, which confer resistance to DDT and neonicotinoids, also confer resistance to azinphos-methyl. This finding is validated by transgenic overexpression of the gene in key metabolic tissues. While we cannot directly implicate azinphos-methyl as a selective agent in the DGRP population, we find that *Cyp6g1*’s range of substrates among insecticides is larger than previously thought, which may explain the strong signature of selection at this locus. This study demonstrates the utility of genomic, transcriptomic, and positive selection scans in developing a more complete picture of a phenotype.

## Supplementary Material

Supplemental Material
