# Supplementary material for: Genomic and Transcriptomic Associations Identify a New Insecticide Resistance Phenotype for the Selective Sweep at the Cyp6g1 Locus of Drosophila melanogaster
Source: G3 (Bethesda). 2016 Jun 15;6(8):2573–81. doi: 10.1534/g3.116.031054 (PMC4978910; doi:10.1534/g3.116.031054)
Supplement: Supplemental Material [file supp_6_8_2573__index.html]

Genomic and Transcriptomic Associations Identify a New Insecticide Resistance Phenotype for the Selective Sweep at the Cyp6g1 Locus of Drosophila melanogaster — Genomic and Transcriptomic Associations Identify a New Insecticide Resistance Phenotype for the Selective Sweep at the Cyp6g1 Locus of Drosophila melanogaster — Supplemental Material 

# Genomic and Transcriptomic Associations Identify a New Insecticide Resistance Phenotype for the Selective Sweep at the *Cyp6g1* Locus of *Drosophila melanogaster*

## Supplemental Material for Battlay *et al.*, 2016

**Files in this Data Supplement:**

- File S1 - Detailed descriptions of all supplemental files. (.pdf, 6 KB)
- Figure S1 - Plot of normalized *Cyp6g1* transcript level from (A) males and (B) females, measured by Huang *et al*. (2015) against azinphos-methyl LD50. (.pdf, 967 KB)
- Figure S2 - Nucleotide variation in exons III and IV of *Ace* in the DGRP, relative to *y; cn bw sp;* reference sequence. (.pdf, 474 KB)
- File S2 - Phenotypes for 178 DGRP lines used in the five GWAS (four single doses and LD50). (.csv, 9 KB)
