# Supplementary material for: Genomic and Transcriptomic Associations Identify a New Insecticide Resistance Phenotype for the Selective Sweep at the Cyp6g1 Locus of Drosophila melanogaster
Source: G3 (Bethesda). 2016 Jun 15;6(8):2573–81. doi: 10.1534/g3.116.031054 (PMC4978910; doi:10.1534/g3.116.031054)
Supplement: Supplemental Material [file supp_g3.116.031054_FileS1.pdf]

S2. Phenotypes for 178 DGRP lines used in the five GWAS (four single doses and LD_{50}).

Figure S1. Plot of normalized *Cyp6g1* transcript level from (A) males and (B) females, measured by Huang *et al.* (2015) against azinphos-methyl LD_{50}. *Cyp6g1-M* alleles are marked in black.

Figure S2. Nucleotide variation in exons III and IV of *Ace* in the DGRP, relative to *y; cn bw sp;* reference sequence. Positions of four common insensitivity substitutions (Menozzi *et al.,* 2004) are marked. The G368A substitution is absent in DGRP lines.
