# Supplementary figures and images for: Genomic and Transcriptomic Associations Identify a New Insecticide Resistance Phenotype for the Selective Sweep at the Cyp6g1 Locus of Drosophila melanogaster
Source: G3 (Bethesda). 2016 Jun 15;6(8):2573–81. doi: 10.1534/g3.116.031054 (PMC4978910; doi:10.1534/g3.116.031054)

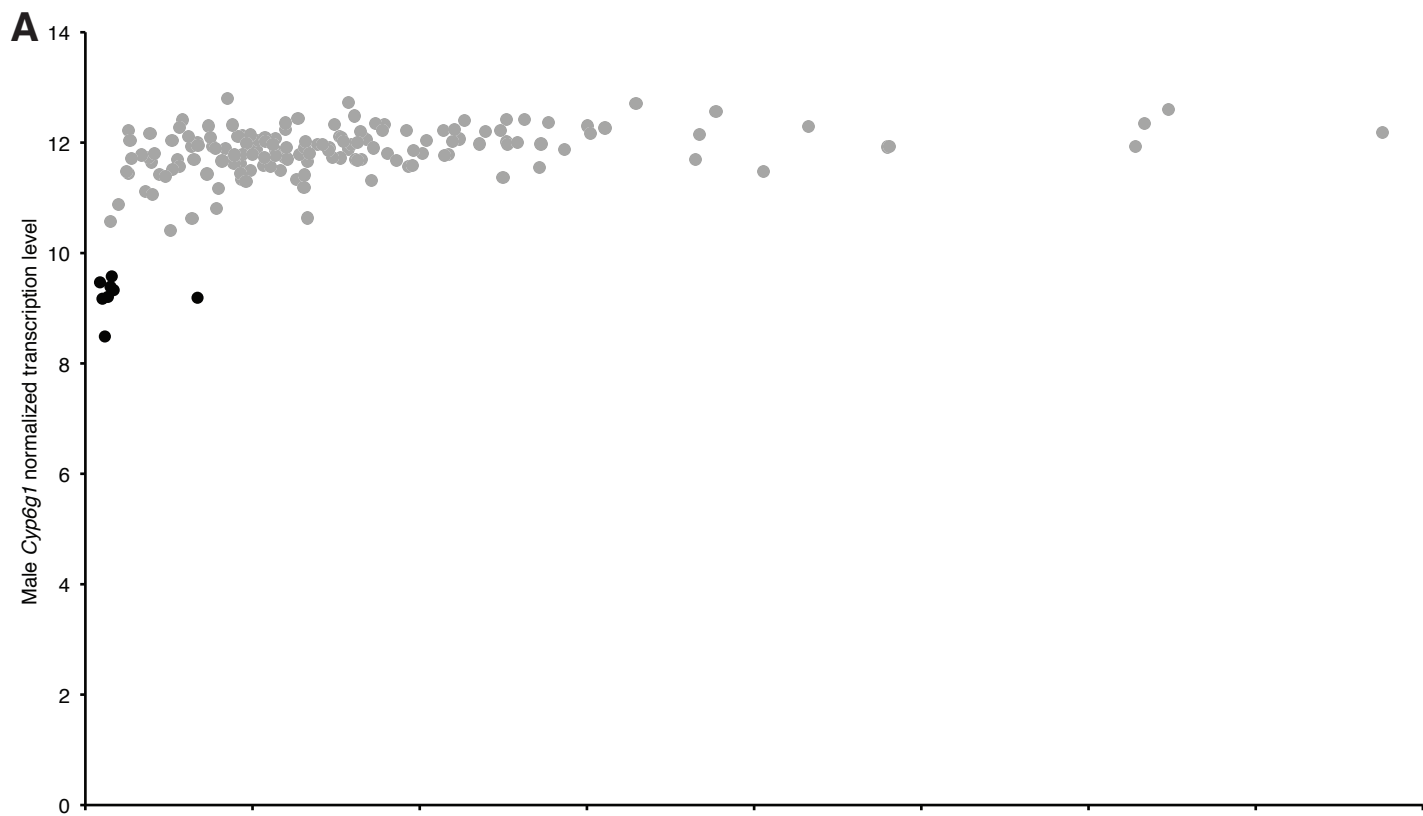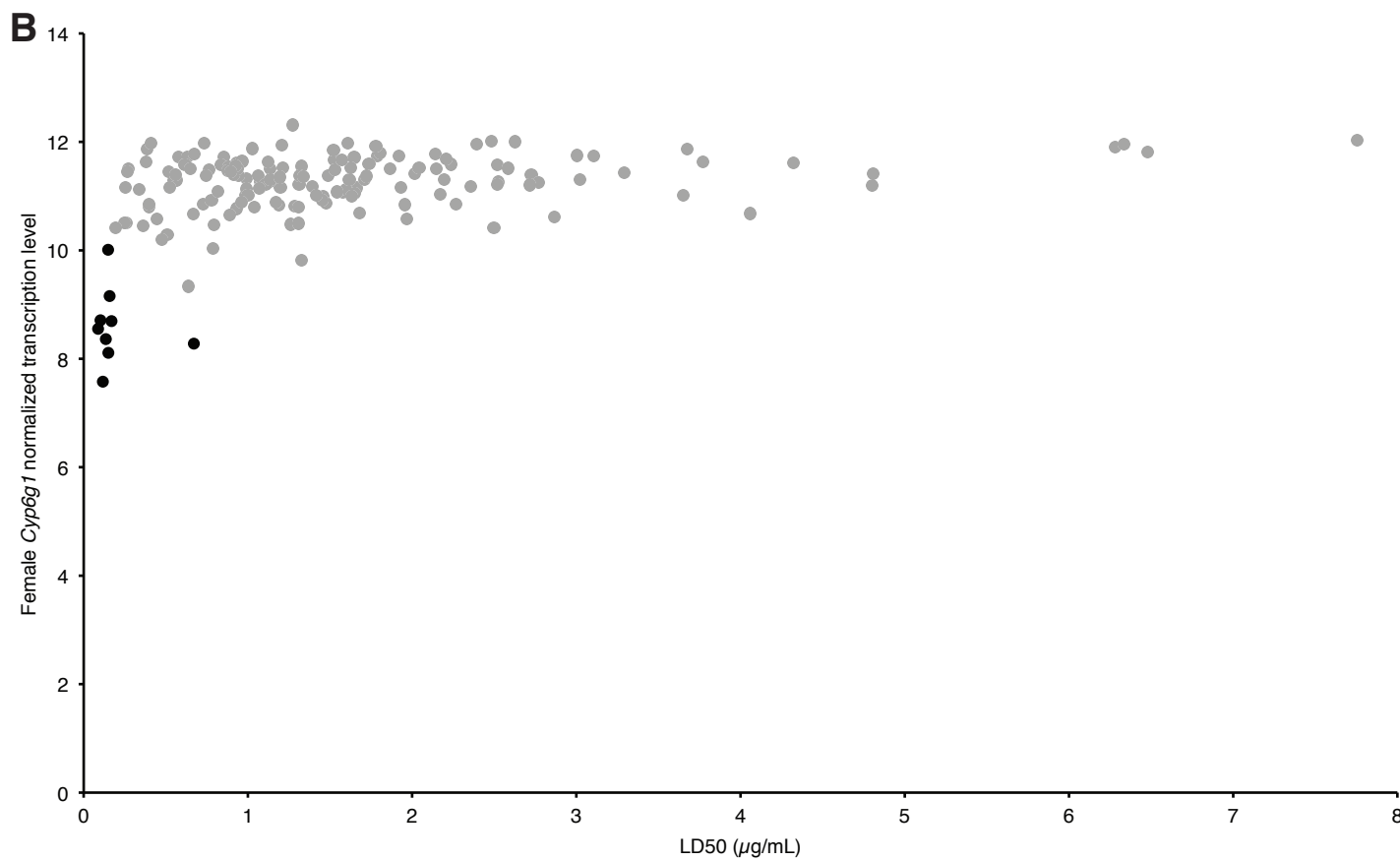

Supplement: Supplemental Material [file supp_g3.116.031054_FigureS1.pdf]

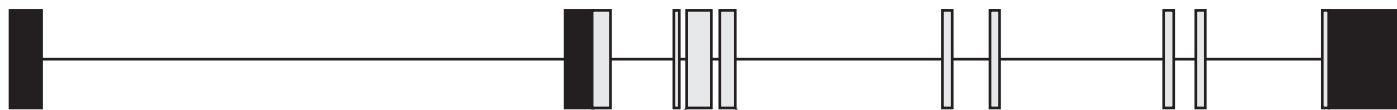

*Acetylcholinesterase*  
3R:13222951-13259517

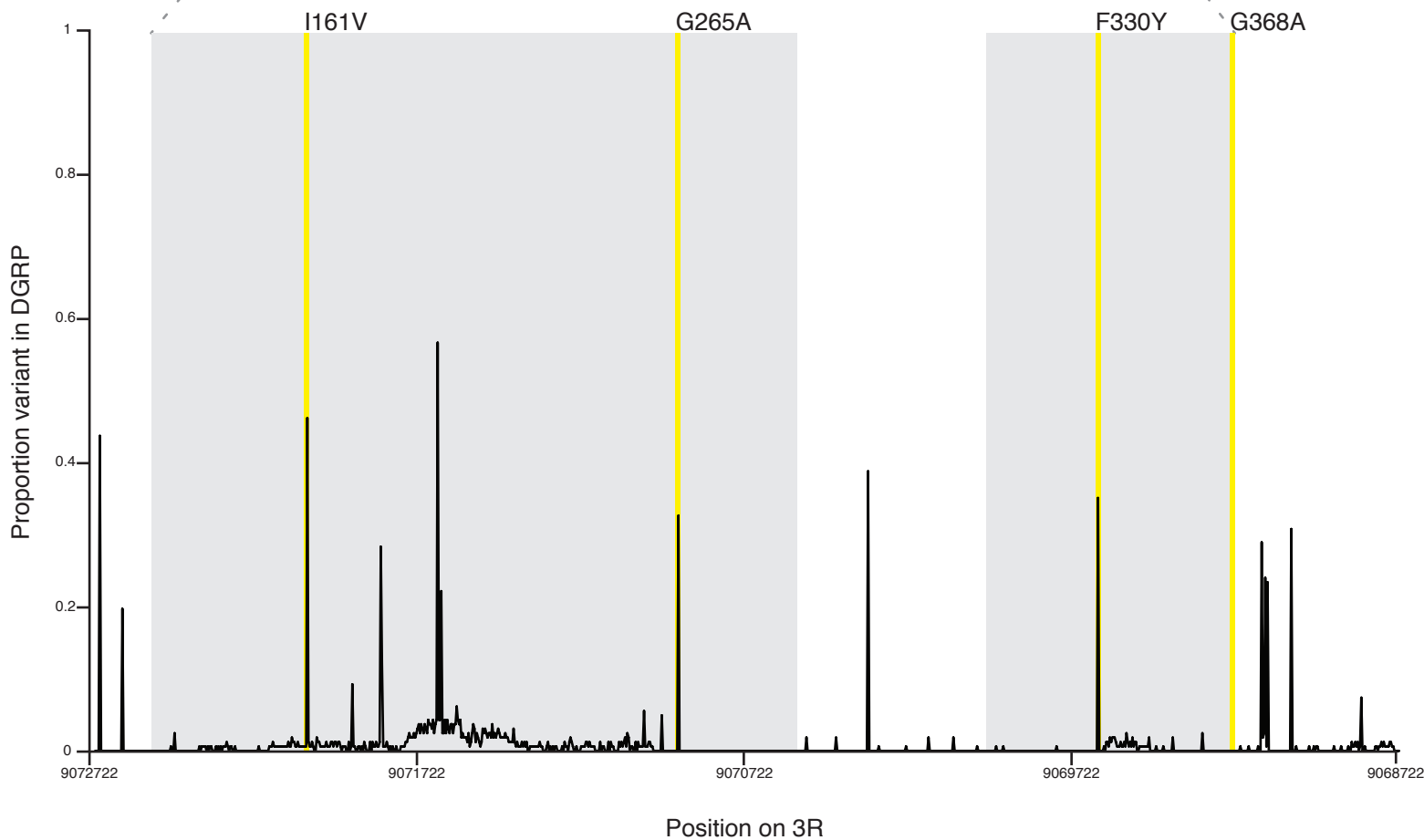

Supplement: Supplemental Material [file supp_g3.116.031054_FigureS2.pdf]
